# A study of diverse clinical decision support rule authoring environments and requirements for integration

**DOI:** 10.1186/1472-6947-12-128

**Published:** 2012-11-12

**Authors:** Li Zhou, Neelima Karipineni, Janet Lewis, Saverio M Maviglia, Amanda Fairbanks, Tonya Hongsermeier, Blackford Middleton, Roberto A Rocha

**Affiliations:** 1 Clinical Informatics Research and Development, Partners HealthCare, 93 Worcester Street, 2nd floor, Wellesley, MA 02481, USA; 2 Division of General Internal Medicine and Primary Care, Brigham and Women’s Hospital, 1620 Tremont Street, Boston, MA 02120, USA; 3 Harvard Medical School, 651 Huntington Avenue, Boston, MA 02115, USA

**Keywords:** Clinical decision support, Knowledge management, Knowledge engineering, Knowledge authoring tool, Rule-based decision support, Knowledge lifecycle

## Abstract

**Background:**

Efficient rule authoring tools are critical to allow clinical Knowledge Engineers (KEs), Software Engineers (SEs), and Subject Matter Experts (SMEs) to convert medical knowledge into machine executable clinical decision support rules. The goal of this analysis was to identify the critical success factors and challenges of a fully functioning Rule Authoring Environment (RAE) in order to define requirements for a scalable, comprehensive tool to manage enterprise level rules.

**Methods:**

The authors evaluated RAEs in active use across Partners Healthcare, including enterprise wide, ambulatory only, and system specific tools, with a focus on rule editors for reminder and medication rules. We conducted meetings with users of these RAEs to discuss their general experience and perceived advantages and limitations of these tools.

**Results:**

While the overall rule authoring process is similar across the 10 separate RAEs, the system capabilities and architecture vary widely. Most current RAEs limit the ability of the clinical decision support (CDS) interventions to be standardized, sharable, interoperable, and extensible. No existing system meets all requirements defined by knowledge management users.

**Conclusions:**

A successful, scalable, integrated rule authoring environment will need to support a number of key requirements and functions in the areas of knowledge representation, metadata, terminology, authoring collaboration, user interface, integration with electronic health record (EHR) systems, testing, and reporting.

## Introduction

Clinical decision support (CDS) is regarded as one of the core functions essential to a modern Electronic Health Record (EHR) [1,2], and the addition of CDS functionality has been a critical step in the evolution of EHRs from a primarily billing and documentation tool to a true patient care and population management tool. Certification of an EHR under the U.S. Department of Health and Human Services’ Meaningful Use criteria also requires some degree of clinical decision support capability [1,2]. A good deal of effort has been devoted to analyzing the effects of implementing new CDS interventions on workflow, practitioner performance and patient outcomes [3,4]. Much less effort to date has been invested in examining the tools that enable the creation and maintenance of the content used by CDS applications.

**Figure 1:**
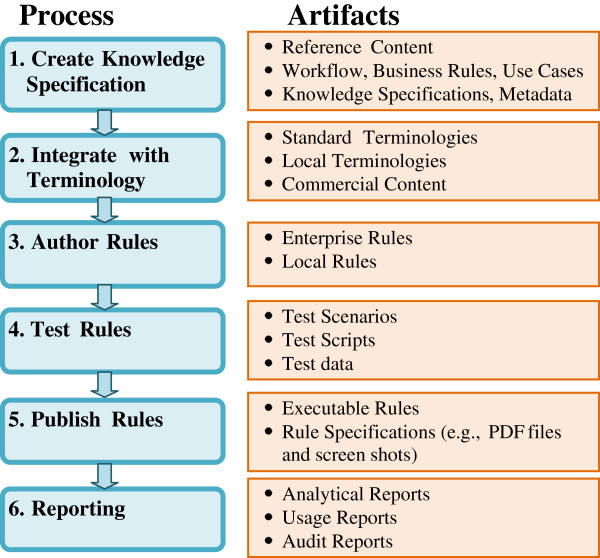
**Major processes for rule authoring.** This diagram outlines six key processes for authoring CDS rules, key artifacts in each process and some associated dependencies. It was developed to assist in defining the requirements for a successful integrated RAE.

Rule authoring environment (RAE) refers to the suite of tools that manage the end-to-end process of creating specifications for rules, integrating with terminology, and authoring, testing, publishing and reporting on those rules. Comprehensive RAEs that touch each step of that overall process are critical to create and maintain decision support interventions and to make CDS rules interoperable and sharable at the enterprise level, regional level, or even national level. Figure 1 shows major rule authoring processes and corresponding knowledge artifacts. Our aim is to examine existing technologies and tools for designing, developing and maintaining content for CDS rules, to study the major requirements for RAEs, and to analyze the gaps to date in fulfilling these requirements.

## Background

The process of converting medical knowledge, which is usually represented as text-based, ‘unstructured’ documents, such as clinical guidelines, to machine executable CDS rules requires the collaboration of clinical Subject Matter Experts (SMEs), Knowledge Engineers (KEs), and Software Engineers (SEs). Shahar et al [5] defined that a gradual conversion process includes four content-representation formats: free text, semi-structured text, semi-formal representation, and a formal representation [6]. Researchers have proposed clinical guideline representation languages and frameworks, such as Arden [7], Asbru [8], EON [9], GEM [10,11], GLIF [12], GUIDE [13], PRODIGY [14], PROforma [15], SAGE [16], and OWL-based approaches [17] for modelling guidelines and protocols in a computer interpretable and executable format [5,18-21]. Even though, as reviewed by Peleg et al [18], these computer-interpretable guideline models have many components in common, they differ in the underlying representation formalisms, decision models, goal representation, use of scenarios, and structured medical actions, and have different intended users and applications. Software tools have been developed to assist users in creating, editing, and interacting with these guideline models. Examples include graphical tools, such as AsbruView [22], Tallis [23], and Protégé [24], and markup-based tools, such as DELT/A [25], GEM Cutter [26], and Degel [27]. There were also other tools developed for capturing the knowledge required to create guideline recommendations [28]. However, none of these existing tools facilitate the entire process flow of transforming free-text content into formal representations and then further into executable rules designed for deployment in a clinical environment. Therefore, standards and mechanisms are still needed to allow these diverse guideline models and tools to be shared between different institutions and software platforms and to be used to transform guidelines into encoded CDS artifacts that can be embedded in real CDS systems [18].

This study focuses on rule authoring environments (RAEs) used primarily to create and maintain rules for common CDS interventions (such as alerts and reminders) implemented in diverse EHR systems. RAEs include not only rule editors used to author rules, but also other ancillary tools needed to span the steps of the rule authoring process as described in Figure 1: creating knowledge specifications, integrating with terminology, authoring rules, testing rules, publishing rules and reporting.

In early efforts at implementing CDS , it was often the case that rules were hard-coded in the EHR system and changes required a significant amount of software development time. In some cases it might take weeks or months before a revised rule was deployed to production [29]. Recent efforts have been made to design rule editors that isolate knowledge base from execution. As reported by Regier et al [29] at Partners Healthcare, the introduction of a rule editor that interfaced with an external knowledge repository was found to greatly reduce that turnaround time and give KEs more visibility of and control over the content. Columbia University has created a knowledge-acquisition tool to allow users unfamiliar with the Arden Syntax to create Medical Logic Modules (MLMs) [30]. It attempts to divorce the entry of decision logic from the underlying procedural code, and is specific to the MLM knowledge representation. Hulse and colleagues [31] developed an XML-based knowledge authoring environment to compose order sets and other structured clinical knowledge documents, though not specifically CDS rules.

### Goals of analysis

Partners HealthCare is an integrated health care system in the Boston metropolitan area, founded by Brigham and Women's Hospital and Massachusetts General Hospital. It also includes several community hospitals, specialty facilities, and other health-related entities. CDS has long been a priority at Partners, and the institution is regarded as one of the major sources of research and development related to CDS [32]. The Knowledge Management (KM) team at Partners develops and maintains a large variety of clinical knowledge content for Partners’ EHRs [33]. KEs on the KM team are usually clinicians, including nurses, pharmacists, and physicians. The KEs serve as a ‘bridge’ between SMEs and SEs, and normally rely on RAEs to author and maintain CDS rules. Along with the evolution of EHRs at Partners, RAEs have been developed at different time periods for multiple purposes and implemented on a variety of platforms. Some of these tools are standalone, while others are embedded within specific EHRs. Some are used to develop a centralized and shared knowledge base, for use in multiple clinical settings and systems, while others are used to build local or application-specific rules. There are no shared patient data models, knowledge representation formalisms, or computer-interpretable guideline models that have been used to design and implement these RAEs. Therefore, such non-interoperable environments limit our efficiency in managing the collections of CDS rules.

We conducted an assessment of the existing RAEs used by Partners’ KM team to author content for CDS rule-based interventions. In this paper, we will describe the role of each tool and identify common and unique features among the tools. We will analyze the advantages and limitations of each tool. From our analysis, we will compile the key requirements and challenges for RAEs. This step is critical to help us identify possible solutions to achieve our goal of developing common CDS rules as well as centralized rule execution services across different systems [34].

## Methods

We identified currently active and supported CDS RAEs used by Partners’ KM team with a focus on the following two categories:

*Reminder Rule Editors*: RAEs for managing clinical reminder rules implementing chronic disease management and general health maintenance guidelines.

*Medication Rule Editors*: RAEs for creating and maintaining rules for medication management CDS, including drug-drug, drug-food, drug-disease, drug-lab, drug-pregnancy interaction, and other drug-related rules.

Reminder rules extend to areas such as women’s healthcare, pediatric care, general adult care, or care for patients who have specific diseases, such as osteoporosis, diabetes, or hypertension. Examples of these types of reminders include overdue immunizations, recommended screening tests such as mammograms, or recommendation for Hemoglobin A1c ordering for diabetes. Examples of active medication rules include warnings for Tylenol dosing in liver disease, ACE inhibitor dosing when creatinine is elevated, or safety warnings for antidepressants in pregnant patients.

For each RAE, we identified the software platform on which the RAE was developed, clinical setting in which the rules are implemented (inpatient or ambulatory), and clinical system in which the tool is embedded. We also identified the scope of rules that are managed by the system and estimated the number of active rules currently managed.

In order to identify strengths and weaknesses of the individual systems, we conducted informal meetings with KEs familiar with the tools to discuss their general experience using these RAEs and the advantages and limitations of these tools. For some technical features, we also consulted SEs. This work was performed as part of an internal review of information system applications and therefore Institutional Review Board approval was not required.

## Results

### Overview of the rule authoring environments

The RAEs at Partners included in this study are shown in Table 1. They are used for different types of CDS rules on different systems and platforms. The rules maintained in each tool are variably implemented for consumption enterprise-wide, for the ambulatory EHR only, or for specific hospital systems.

**Table 1:** Different RAEs and their characteristics^*^

**RAE**	**Setting**	**Clinical System**	**Scope**	**Est. # of Rules**^**ξ**^
**REMINDER RULE EDITORS**				
**Reminder Editor** (1st generation)	Ambulatory	Standalone	Used by KEs for entering and editing reminder rules	356
**Reminder Editor** (2nd generation)	Ambulatory	Embedded in an ambulatory EHR	Used by KEs for entering and editing reminder rules	356
**Enterprise Rule Services** (3rd generation)	Ambulatory	Standalone; service-oriented	Used by KEs for entering and editing diverse clinical decision support rules	120
**MEDICATION RULE EDITORS**				
**Drug-Drug Interaction (DDI) Editor**	Ambulatory and inpatient	Standalone; web-based application	Used by KEs for entering and editing DDI rules	2800
**Renal Drug Dosing** (Nephros)	Ambulatory and inpatient	Embedded in an inpatient CIS. CDS rules are maintained in a specific EHR, but are used at the enterprise level by other systems	Used by KEs for entering and editing rules to offer substitute drugs or adjust dose or frequency list for a drug for renally impaired patients (e.g., based on a particular Creatinine value)	352
**Geriatric Drug Dosing** (Gerios)	Ambulatory and inpatient	Embedded in an inpatient CIS. CDS rules are maintained in a specific EHR, but are used at the enterprise level by other systems	Used by KEs for entering and editing rules to offer substitute drugs or adjust dose or frequency list for a drug for geriatric patients, based on a particular age value	244
**Neonate Pediatric Drug Dosing**	Inpatient	Embedded in a medication concept editor	Used by KEs for entering and editing rules to offer substitute drugs or adjust dose or frequency list for a drug for pediatric patients	1000
**Food Drug Interaction**	Ambulatory and inpatient	Embedded in a medication concept editor	Used by KEs for entering and editing FDI rules	200
**Duplicate Therapy**	Ambulatory and inpatient	Standalone; web-based application	Used by KEs for entering and editing rules to detect potential drug duplications	190
**Medication Rule Editor**	Ambulatory	Embedded in an ambulatory EHR	Maintains its own set of medication rules, including drug-disease, drug-pregnancy, drug lab, drug-group, group-group interactions	2300

^*^The platform of Medication Rule Editors is largely based on Cache and Visual Basic. The platform of Reminder Rule Editors varies. 1st generation is Microsoft Access-based, 2nd generation is Cache based, and 3rd generation is Java-based.

^**ξ**^Medication rule editors as of February 2009, Reminder rule editors as of February 2012.

### Diversity and unique features of the rule authoring environments

**Figure 2:**
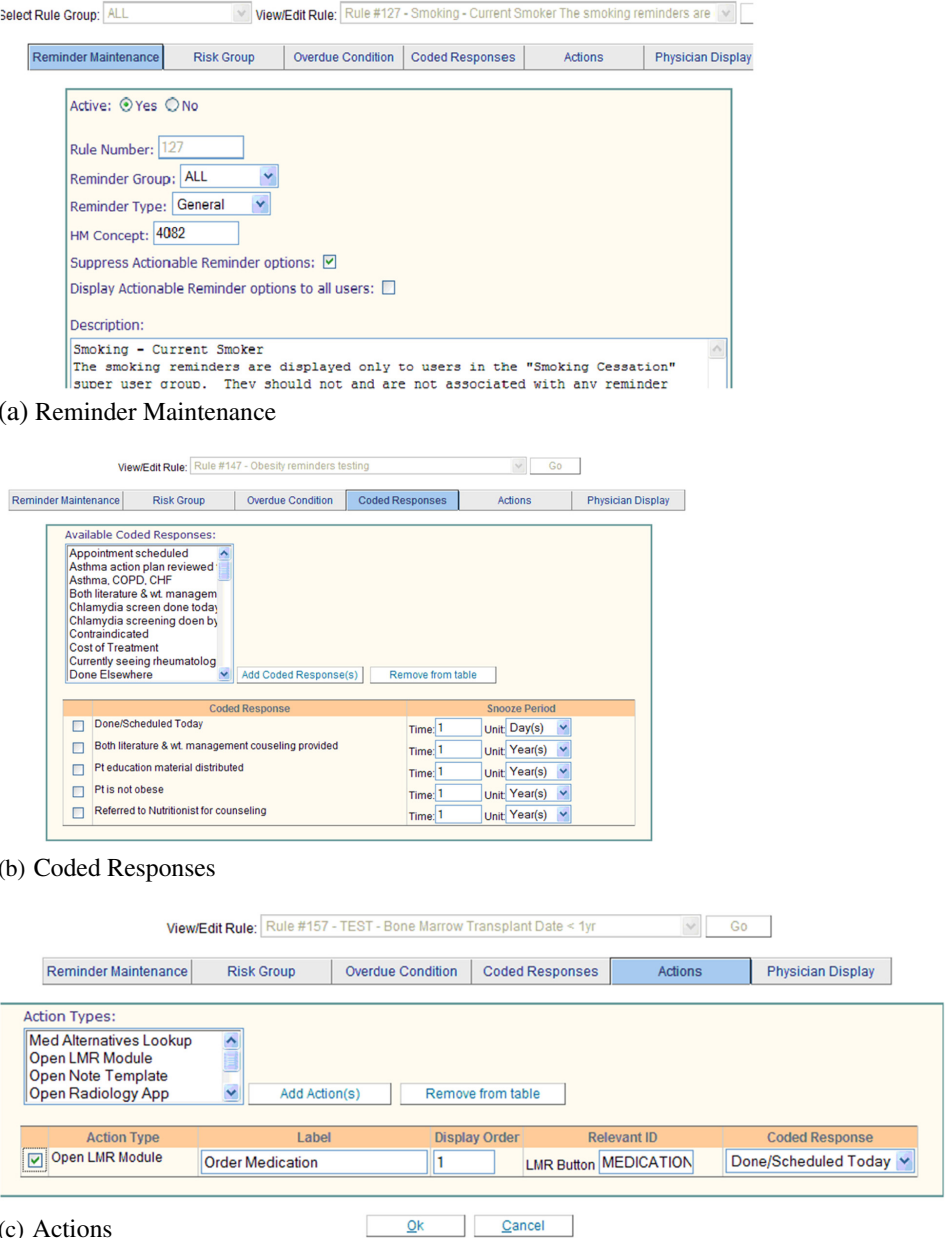
**Reminder Editor.** The Reminder Editor allows users to add a new rule or view/edit existing rules. It has six tabs and this figure includes screen shots of three of the six tabs of the Editor. 1) Reminder Maintenance (Figure 2a) records the metadata and a description of a rule. 2) The Risk Group tab defines specific risk group primitives (such as age, allergy, and disease). 3) Overdue Condition evaluates the most recent data of a test (e.g., Hemoglobin A1c) performed on the patient and determines if the test is due or overdue. 4) Coded Reponses (Figure 2b) are predefined clinician recommendations or actions to be linked to the reminder. Each coded response may have a snooze period associated with it. Snooze periods allow the clinician to defer the follow-up actions for a period of time. 5) Actions (Figure 2c) are commands that will automatically open other system modules or places within LMR. This will allow the clinician to easily implement a coded response reminder recommendation for orders, medications, radiology tests, and so on. 6) Physician Display defines the exact reminder message to be displayed in the patient’s electronic medical records and also defines references (e.g., guidelines) for this reminder.

In addition to a variety of platforms, scopes, and clinical systems, the various editors also differ widely in design and workflow process. The Reminder Editor, which has been discussed in a previous paper [29], is a complex editor designed to support authoring of diverse reminders. Compared to the medication rules editors, which are designed for more specific CDS interventions, the Reminder Editor presents more decision points and options for the KE to choose from, as shown in Figure 2. It assists KEs to manage metadata and define rule logic (e.g., risk group, overdue conditions, coded responses, and message to clinicians receiving the reminder). It also allows KEs to specify links to guidelines, supporting literature, and other reference content.

**Figure 3:**
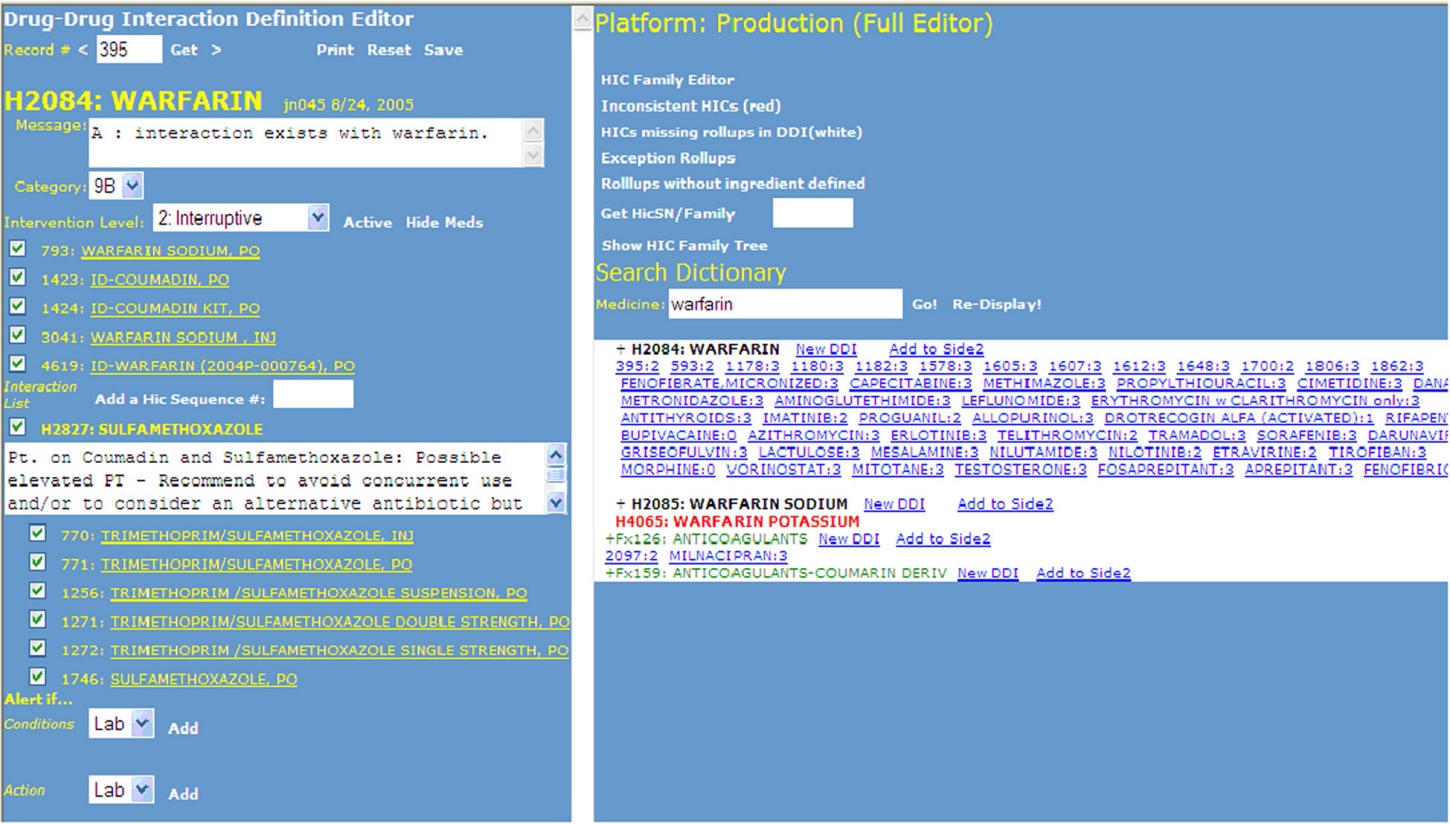
**DDI Editor (interaction between Warfarin and Sulfamethoxazole).** On the right pane, a user can search for existing DDIs or add a new DDI by entering a drug name (e.g., Warfarin) in the Medicine search field. The search returns existing DDI records organized by Warfarin concepts coded by the First Data Bank Hierarchical Ingredient Code Sequence Number (HIC-SEQN). For example, H2084 is the HIC-SEQN for Warfarin. In the top right panel, the Editor provides some links to HIC_SEQN and Partners local drug dictionary. 395:2 represents a DDI record for Warfarin, where 395 is a system-generated DDI identifier and 2 is the level of the intervention (i.e., interruptive). The other two levels of intervention are 1- dead stop and 3 - non-interruptive. A text box appears when the user hovers over 395:2 and shows more information about the DDI. To view the details of a DDI, a user can click on one of the DDI records or type the DDI identifier in the Record # box in the top left panel of the DDI Editor. On the left panel, the Editor displays the details of a DDI, including message, locally defined category, intervention level, drugs in Partners drug dictionary that are mapped to the HIC-SEQN, conditions and action. The user can modify existing DDI or define a new DDI using the user interface on the left panel.

The Drug to Drug Interaction (DDI) editor is an example of a tool that is designed for a specific workflow: authoring medication interaction rules. A DDI rule usually consists of two medication concepts (either an ingredient or a drug family), intervention level (e.g., dead stop, interruptive, or non-interruptive), message, conditions (e.g., if a Potassium lab comes back > 3 within 24 hours, a DDI associated with this condition will fire), and actions (e.g., order lab test when a particular DDI fires). It has a well-defined set of input requirements and generates rules without complicated branching logic, and thus has a relatively fixed user interface, as shown in Figure 3.

While the inputs for entering a DDI rule are relatively well defined, complexity is introduced in the tool by the need to manage both individual drugs and drug families. Since a DDI rule is often based on drug ingredient or drug family, the editor needs to facilitate a complicated process to manage and validate individual drugs that belong to the same family or have the same ingredient, but have multiple trade names and dosage forms.

**Figure 4:**
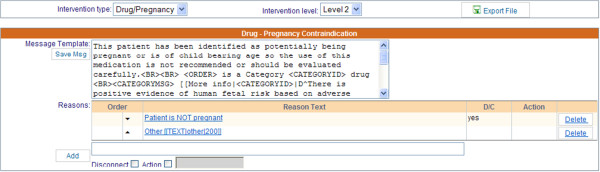
**Medication Rule Editor in an ambulatory EHR.** Rule editor used in ambulatory EHR to author drug-disease, drug-pregnancy, drug lab, drug-group, group-group interactions.

The Medication Rule Editor embedded in the Partners ambulatory EHR maintains its own set of medication rules, including drug-disease, drug-pregnancy, drug-lab, drug-group, and group-group interactions. The editor has a user interface that KEs find intuitive (Figure 4), similar to the Reminder Editor, but very different from the other medication rule editors, such as the DDI editor.

### Major limitations of current systems

From our analysis of the various active RAEs in use across Partners, several limitations of the current distributed system became apparent, as described in the following.

#### Isolated

As noted in Table 1, most editors are embedded in either inpatient/ambulatory EHR systems or terminology editors and do not interface with a centralized knowledge repository. This can lead to overlaps or conflicts in rules applicable to a given clinical scenario. For instance, one set of rules may define inclusion criteria for diabetic patients based on lab values, while another may define inclusion criteria based on problem list entries. An integrated editor and shared repository with robust reporting capabilities would facilitate the process of identifying and resolving rules that conflict in this way. However, it would not solve the problem of rule collision entirely. For example, in the current system, renal adjustment of drug dosing is maintained in the Renal Drug Dosing (“Nephros”) editor, while age-based dosing is maintained in the Geriatric Drug Dosing (“Gerios”) editor. If a medication concept is involved in both types of rules, the KE needs to manually decide which rule to use based on input from SMEs. In this case, it is the specific patient scenario, geriatric patients who are also renally impaired, that leads to conflict rather than inconsistent rule logic. An integrated editor may assist KEs in identifying and resolving potential collisions, but would not eliminate occurrence of these scenarios.

#### Nonsharable

The rules maintained in each tool may be used at the enterprise level, but may be specific to ambulatory EHRs only, or to specific hospitals or systems. Medication rules that are created by the DDI editor, Nephros, and Gerios are used at the enterprise level by other systems, but those created by the Medication Rule editor are specific to the ambulatory EHR. These 2,300 rules are likely applicable to other practice environments, but are not available unless duplicated manually. The “silo” nature of the current system, with lack of enterprise-level availability of all rules, is not just a result of having multiple authoring environments. It is also a result of having separate knowledge repositories and execution engines relying on different knowledge representations. The resulting complexity leads to inefficiency and duplication of effort.

#### Nonstandardized

While physical sharing of the separate rule repositories could theoretically be implemented in their current format, this could quickly become a complex task due to the diversity of representation models and terminologies. Most rules are expressed using local dictionaries and using non-standardized knowledge representations. Bi-directional transformation of rules between separate environments and maintenance of inter-system concept mapping is inefficient and untenable as a long-term strategy. We are currently evaluating the feasibility of using standard terminologies to encode data elements in the rules (e.g., NDF-RT [35] and RxNorm [36] concepts for medication rules); however, terminology mapping and integration will still be a significant effort. An integrated editor using a common rule representation model, patient data model, and standard terminologies would assist with this effort at the authoring level. Although it would not eliminate the need for data mapping at the implementation level, it would facilitate future development of an enterprise-level rule engine or execution service.

#### Nonextensible

The current RAEs are not well-structured to accommodate the future complexity of knowledge representation, because they were built to support the current state of the rule content, which is primarily if-then logic and some of the user interfaces were customized to match Partners’ local terminology (see Figure 3). For instance, genomic data and personalized medicine will likely become clinically significant in the foreseeable future [37], but could not be accommodated by any of the existing specialized RAEs. Incorporating this new content, such as drug-genome rules, would require modification of an existing editor, or development of a new editor. Rule logic is likely to get more complicated. For example, an extensible RAE should be able to support rule chaining or inferencing, which starts with some data and uses inference rules to extract more data until a conclusion is reached. Having a flexible, integrated authoring environment that is not content specific would require an underlying extensible knowledge representation model that is able to accommodate the present and future knowledge. Such an environment should also be able to provide a shared framework for terminology integration, testing, reporting, lifecycle management, and other non-content specific tasks.

#### Disjointed

None of the RAEs have native support for the front-end process of transforming guidelines into CDS rule specifications, or for soliciting the input of SMEs in design and validation of these specifications. At Partners, a collaboration platform is used to enable this process; however, the collaboration platform is not integrated with any of the RAEs, thus once the rule specification is validated and approved by the SMEs, the KEs must then manually transfer the rule details to the applicable RAE. The KEs have the additional overhead of maintaining the provenance and metadata that associates the validated rule specification in the collaboration platform to the implemented rule(s) in the RAE.

#### Costly

Developing and maintaining separate RAEs requires the efforts of specialized KEs and SEs, all of which are scarce assets at most institutions. We were able to identify several cases of workarounds and ‘hard-coding’ used to overcome current editor and knowledge representation deficiencies. It is easy to imagine a scenario in which the primary developer or expert user of a specific tool leaves the organization and the knowledge about the tool’s intricacies is lost. It could be argued that having specialized editors and KEs could make the system more efficient by limiting the set of skills and training needed to use the system. For example, KEs primarily responsible for encoding drug-drug interaction rules may want a streamlined interface that allows them to enter their data quickly, and may not need to know about reminder rules or editors. Even in this scenario, having a generic environment built around a specialized editor will support maintenance of shared processes such as terminology integration, testing, reporting, and lifecycle management that apply to all rules without sacrificing efficiency.

## Discussion

In this paper, we have reviewed a set of clinical rule authoring environments used by Knowledge Engineers at Partners, which manage more than 7,000 CDS rules. Currently, there is no single RAE that meets all requirements for all types of rules, and there are numerous drawbacks of the current distributed system. We suspect that many other large-scale institutions with homegrown decision support systems went through a similar gradual and piecemeal process of developing applications and tools for CDS as the Partners system. Our findings are of interest to set the requirement for better management as institutions race headlong into CDS for a mature EHR and Meaningful Use.

### Critical success factors

Based on interviews with KEs and SEs and our analysis of the rule authoring process, we can identify a number of critical success factors and key functions that need to be supported in order to establish a RAE in a robust, scalable and extensible way. The functions described in this section align with the processes in Figure 1 and the requirements in the next section.

#### Formal knowledge representation and standards

The centralized, distributed rule service approach requires rules represented and stored in a formal and standard representation to be deployed to diverse EHRs. Current RAEs use ad-hoc approaches to rule representation due to lack of standards when they were built, combined with the desire to address specific site needs or content areas quickly. Usually, such legacy tools are localized in a healthcare organization without connection with other information systems and without interfaces to exchange information between them. Changing this situation is a challenging task that requires widely accepted open standards and interface protocols, supported by robust implementation tools.

Previous studies have proposed multiple knowledge representation formalisms and models to represent clinical practice guidelines [18-20], as reviewed in the background section. These models are used to represent knowledge elements contained in guidelines, such as plans, goals, actions, and decisions, as well as temporal relationships (e.g., sequential, parallel, or cyclical) and constraints between elements. In contrast, current RAE representation models are typically only able to express limited metadata with relatively simple triggering conditions, coded responses, and actions. An ideal knowledge model should be capable of expressing different types of clinical knowledge, including complex relationships and constraints. Future work is needed to validate more expressive guideline representation languages and frameworks, particularly their effective integration with EHR systems.

#### Metadata support

Extensive metadata support leveraging standard terminologies is essential to assist KEs in the curation of large sets of interdependent CDS artifacts. For example, there might be as many as a hundred CDS artifacts for the management of hyperlipidemia that take account of multiple diagnosis combinations, multiple laboratory results, as well as multiple medication combinations and titration considerations. It is essential that the KE is able to identify these as a relevant set and visualize them in a manner that facilitates detection of gaps, inconsistencies and/or errors.

#### Terminology integration

Medication terminology serves as a foundation for DDI rules. Our local medication terminology, which is used to encode medication orders in Partners’ EHRs, has a relatively simple (non-hierarchical) structure. However, each local medication concept is manually mapped to a First Data Bank (FDB) [38] ingredient, enabling the identification of classes of medications that share the same ingredients. KEs can look up and retrieve these proprietary medication concepts and classes using a RAE that is integrated with the local medication terminology database. The current DDI editor is rudimentary and requires an experienced user to select and manage the appropriate medication concepts and their classes. For reminder rules, the RAEs are not fully integrated with terminology services and KEs have to manually lookup and transcribe the relevant terminology concepts and classes. If a well-structured terminology is available, then terminology management should ideally be separated from the rule development; terminology services should provide a seamless integration of terminology into the rule authoring process.

There are specific requirements and challenges for terminology services to fully support an integrated RAE. Currently at Partners, relevant terminology services are maintained by different teams. One team covers services for problems, diagnoses, and procedures. Another team covers services for medications and allergies. Laboratory test codes are provided by yet another team. In this scenario, the terminology content, technical infrastructure, and application development platforms are managed independently. In order to provide full support for an integrated RAE, these various services need to be efficiently integrated.

Another challenge worth mentioning is the management of terminology classes (subsets), including problem classes, which are used to group clinical findings that define a patient’s clinical state (e.g., if the patient has diabetes mellitus), and medication classes, which are used to group medications that have the same therapeutic effects, or the same set of ingredients. The problem classes and the medication classes are created and managed using different editors. These editors allow users to view and navigate standard terminologies and local extensions created by Partners. While automated processes have been developed to detect new candidates for the problem classes, the maintenance of the subsets is not fully automated. Some classes have constraints (e.g., a class for total hysterectomies that excludes partial hysterectomies as this class is used to suppress PAP smear reminders) and SME review and approval is always required before classes are released.

#### Collaboration support

The RAE should be able to support and facilitate the collaborative, iterative, and transparent processes amongst clinical SMEs, KEs, and SEs [39]. However, today at Partners, the collaboration environment is not integrated with the RAEs, so documents and specifications vetted by SMEs (e.g., free-text or intermediate representation of rule logic) are stored, maintained and shared separately from the RAE as Microsoft Word or Excel documents. Only a few RAEs, including the Reminder editor, provide links to the guideline, literature, or reference specification. Recently, we have created structured and coded rule specifications using XML and XSLT technologies [31], but these have not yet been incorporated into the existing RAEs. A robust RAE should allow KEs to create free-text or intermediate semi-structured representations of rule logic, allow SMEs to comment on and adjust the logic, convert them to a formal, executable representation, and then submit it to developers to integrate into the receiving application for execution, all in an integrated and systematic method.

The RAE should be able to support the efficient collaboration between medical terminologists and KEs. When only local concepts are used in rules, KEs do not need much assistance from terminology engineers (TEs). As standard concepts, concept classes, and various mappings are incorporated, there is a greater need for support from TEs, SMEs, and Informaticians. It is often the case that new CDS artifacts require the development of new concept classes to support the CDS concern. KEs should focus on the rule logic and consult with the appropriate specialists as needed.

#### User interface

A sophisticated graphical user interface is critical for user friendly RAEs. It should not only support more efficient and effective user interaction, but also address the individual end user (e.g., KE, SME, or SE) needs and expectations. One critical success factor of the Reminder editor is that it provides (primarily for KEs) a simple and intuitive user interface that abstracts the complexity and subtlety of the underlying clinical knowledge. Some commercial or open-source products use traditional rule logic representations and artifacts such as if-then rules (with specific syntax), decision tables, and decision trees. Recent efforts at Partners are attempting to centralize rule development and maintenance, and to eventually share these artifacts across different EHRs using a Java-based commercial product for rule authoring and execution [40]. This commercial product employs object-oriented modeling techniques to explicitly specify the semantics and structures of clinical information needed for the rules. However, it uses traditional CDS artifacts such as if-then statements and decision tables, which are easy to understand and manipulate by SEs, but not intuitive for clinically trained KEs who usually have limited technical background and programming skills. An intuitive UI can streamline rule development for KEs through the use of well-defined templates for managing metadata and defining rule logic (e.g., risk group, overdue conditions, physicians’ coded responses, and message) in a way that separates the user interface from the underlying knowledge representation needed for rule execution.

#### Integration with EHR systems

Although there are multiple existing guideline execution engines that allow the enactment of clinical guidelines in a semi-automatic or automatic fashion, none of them is actually used in daily clinical practice. A major challenge of the integration task is lack of an underlying common EHR information model, which allows data defined in a guideline model to be mapped to existing EHRs [21].

At Partners, although several editors (such as Reminder Editors, Nephros, and Gerios as shown in Table 1) have been integrated with EHR systems, they are part of the specific systems and therefore not portable. A RAE should adopt a formal information model that represents patient data required in the rules. Regarding the system architecture, the preferred RAE should be integrated with EHR systems, for example, using a modular, service-oriented architecture, so it will be more easily maintainable, portable, and scalable. It should be able to interact with EHR systems, for example, via a set of interfaces and services (e.g., the patient data service that retrieves the appropriate patient data). It should also support testing and reporting using real EHR data as described below. The RAE should adopt standard open protocols and tools when they are available.

#### Testing

A dedicated testing environment and integrated lifecycle management process is essential to track rules from editing to testing to production. The Partners rules reviewed in this study are published to a Quality Assurance (QA) environment for testing, and then moved to Production, often manually. This increases the chance that errors will be introduced in the content with each transition. Further, developing sufficient test patient data and concrete test cases are time-consuming tasks, which require a significant amount of lead time and resources. Given the rule logic and parameters, it is technically feasible and desirable for a properly architected RAE testing system to generate all the test cases necessary to verify the rules.

Testing should be performed in controlled and iterative manner. Testing should verify both the logic of the rule itself and also functionality of the rule in the CDS environment, such as redundancy and overlapping rule logic, missing conditions and actions, conflict, and availability of data in EHR systems [41]. Initial testing may focus on individual or small sets of rules and then be expanded to the full set of rules. This helps to isolate and resolve specific issues or unexpected results. Once the rules are executing as expected, integration and user acceptance testing can be initiated. Once the test process is working consistently, the team should look for opportunities to automate or streamline the process so it can be more easily repeated as the rules are refined.

#### Reporting

Reporting is critical when maintaining rule sets. Most RAEs cannot currently generate reports. Reports for medication rules are generated separately using another user interface connected to the rule repository. The Reminder editor does not support reporting directly from the tool itself, so reports are managed separately in an Microsoft Access database. Robust reporting is needed in order to analyze existing rules and dependencies, usage, and to audit performance and maintenance.

### Requirements for a successful integrated RAE

We have defined the rule authoring workflow as shown in Figure 1. Based on our analysis of the current existing rule authoring environments at Partners and discussion of critical success factors, we created following requirements for each of the processes in Figure 1.

Process 1: Create Knowledge Specifications

Includes reference content, knowledge specification, use cases, workflow, business rules, metadata, etc.

✓ Store reference content and knowledge specifications persistently in a central repository.

✓ Reuse and share existing content and avoid redundancy.

✓ Support communication and collaboration across different teams.

✓ Support metadata tagging to facilitate searching.

✓ Standard process for rule governance and lifecycle management and versioning.

Process 2: Integrate with Terminology

Includes local terminology, commercial content, standard terminology, and value sets.

✓ Ability to handle terminology updates in a timely fashion.

✓ Ability to detect rule impact (be able to triage as no, minor or major impact on rules).

✓ Ability to search, navigate and select terminologies and concepts (e.g., terminology browser) directly from user interface.

✓ Display concept names in the rules to make the rules readable and understandable and store the concept codes for the purpose of rule execution.

✓ Ability to navigate semantic relationships between concepts.

✓ Ability to define, display and search value sets or subsets of related terms.

Process 3: Author Rules

Includes local rules and enterprise rules.

✓ Central maintenance and coordination of content.

✓ Maintain and manage rules without dependence on software development resources.

✓ Life cycle management and versioning.

✓ Ability to check to duplicate or overlapping rule logic.

✓ Ability to construct rules based on templates.

✓ Ability to construct rule artifacts such as decision tables and rule flows.

✓ User-friendly features, such as form fields (e.g., check boxes, pick lists, and text boxes), drag and drop functionality, intuitive advance searching, and help menus.

Process 4: Test Rules

✓ Allow rule logic to be validated independently from the upstream or downstream services and applications.

✓ Eventually integrate with upstream and downstream services and applications in order to perform integration and user acceptance testing activities.

✓ Develop and maintain test patient data to support the rule logic.

✓ Identify redundancy and overlapping rule logic.

✓ Identify missing conditions and actions.

✓ Identify conflicts with existing rules.

Process 5: Publish Rules

✓ Rules need to be made available for execution within production clinical information systems.

✓ Rule specifications must be visible to users at enterprise level.

✓ Executable representation must be available in human readable format for review by SMEs and other business people.

✓ Rules must be publishable to testing and runtime environments without requiring manual re-entry.

Process 6: Reporting

✓ Analytical reports to identify conflicting rules, incomplete rule coverage or rule redundancy.

✓ Comparison reports to identify when rules are dependent on each other, so they can be tested and managed together.

✓ Usage reports to specify which applications consume the rules, so that future changes can be communicated to the relevant teams.

✓ Audit reports to track rule changes, who made the changes and why the changes were made.

✓ Execution reports to track frequency of individual rule execution within the clinical systems and rates of clinician acceptance or rejection of recommendations.

### Recent development

More recently, Partners has developed a multi-layered knowledge representation framework to structure guideline recommendations for implementation in a variety of CDS contexts [42]. This framework has adapted standards recommended by HL7 [43], Healthcare Information Technology Standards Panel (HITSP) and elsewhere, to create a formal information model that describes clinical data required in the rules, and data are encoded using standard terminologies (e.g., SNOMED [44], LOINC [45], RxNorm [36]). An ongoing effort has been made to centralize rule development and provide CDS services across the enterprise.

### Study limitations

A major limitation of the current analysis is that the environments analyzed are limited to the Partners system. A large number of institutions rely on vendor tools for decision support, which vary widely in design and features [46]. Therefore, we cannot completely generalize our findings to other institutions. We also limited our study to RAEs in the areas of reminders and medications CDS. This analysis does not include tools for other types of CDS interventions, such as order sets, templates and infobuttons. The tools in place at Partners all currently use local terminologies and local, non-standard rule representations. We did not analyze in depth the effort or additional tools which may be needed to facilitate translation to and from standardized terminologies. There are currently gaps in standards for rule representations that need to be more fully addressed in order to incorporate them into an integrated RAE. Recommendations from these efforts will likely inform requirements and specifications for future RAEs.

## Conclusions

Most existing RAEs at Partners limit the ability of the CDS interventions to be standardized, sharable, interoperable, and extensible. We have identified key principles to consider in the design of an integrated RAE and have discussed major requirements and challenges in the areas of knowledge representation, metadata, terminology, authoring collaboration, user interface, integration with EHRs, testing, and reporting.

## Competing interests

The authors (LZ, NK, JL, SM, AF, TH, BM, and RR) declare that they have no competing financial interests, including patents, reimbursements, fees, funding, salary, stocks or shares from any organization that may in any way gain or lose financially from the publication of this manuscript. The authors also declare that they have no non-financial competing interests. Funding for this work was provided internally by Partners HealthCare. All authors are affiliated with Partners HealthCare.

## Authors’ contributions

LZ conceived of the study, participated in the design of the study and its coordination, and drafted the manuscript. NK assisted in data analysis and helped to draft the manuscript. JL, SM, and AF assisted in data gathering and analysis. JL, TH, and BM helped draft the manuscript. RR participated in the design and helped draft the manuscript. All authors read and approved the final manuscript.

## Pre-publication history

The pre-publication history for this paper can be accessed here:

http://www.biomedcentral.com/1472-6947/12/128/prepub
